# ToxTeller: Predicting Peptide Toxicity Using Four Different Machine Learning Approaches

**DOI:** 10.1021/acsomega.4c04246

**Published:** 2024-07-11

**Authors:** Jen-Hung Wang, Ting-Yi Sung

**Affiliations:** Institute of Information Science, Academia Sinica, Taipei 11529, Taiwan

## Abstract

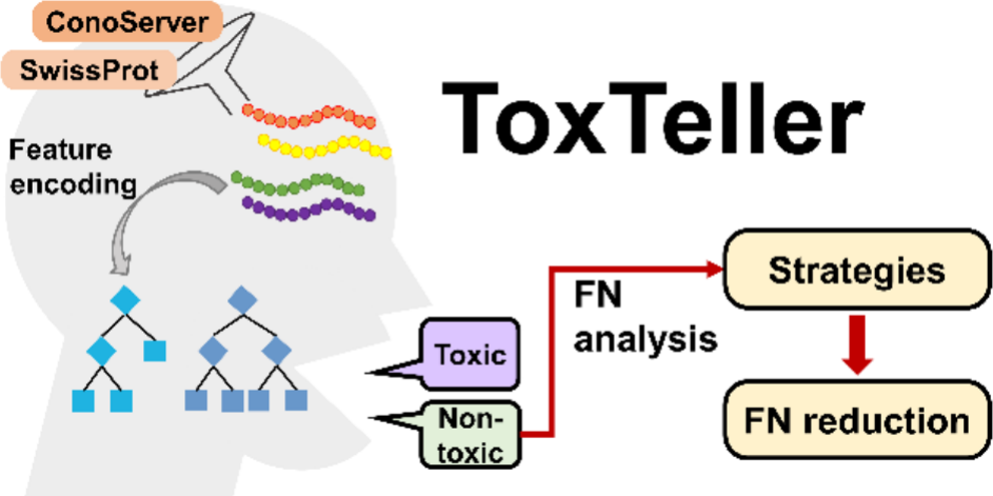

Examining the toxicity of peptides is essential for therapeutic peptide-based drug design. Machine learning approaches are frequently used to develop highly accurate predictors for peptide toxicity prediction. In this paper, we present ToxTeller, which provides four predictors using logistic regression, support vector machines, random forests, and XGBoost, respectively. For prediction model development, we construct a data set of toxic and nontoxic peptides from SwissProt and ConoServer databases with existence evidence levels checked. We also fully utilize the protein annotation in SwissProt to collect more toxic peptides than using keyword search alone. From this data set, we construct an independent test data set that shares at most 40% sequence similarity within itself and with the training data set. From a quite comprehensive list of 28 feature combinations, we conduct 10-fold cross-validation on the training data set to determine the optimized feature combination for model development. ToxTeller’s performance is evaluated and compared with existing predictors on the independent test data set. Since toxic peptides must be avoided for drug design, we analyze strategies for reducing false-negative predictions of toxic peptides and suggest selecting models by top sensitivity instead of the widely used Matthews correlation coefficient, and also suggest using a *meta*-predictor approach with multiple predictors.

## Introduction

Peptides have the advantage of high specificity, high efficacy, safety, and relatively low production cost, and are thus potential therapeutics for cancers and other diseases.^1,2^ Peptide-based vaccine and drug discovery have received increasing attention.^3,4^ When evaluating peptides as therapeutic peptide candidates, in addition to considering their immunogenicity and stability, their toxicity must also be examined.^5^ However, conducting experiments to investigate peptide toxicity is costly and time-consuming. In contrast, it is more efficient and economical to use machine learning (ML) to predict peptide toxicity. In the past two decades, peptide toxicity prediction has attracted much attention in different applications. For example, to design 5–20 peptides of length 15–35 for a neoantigen vaccine, Fang et al.^6^ present a bioinformatics workflow that includes toxicity prediction for the designed peptides using an in-house program.

Several predictors have been published for protein or peptide toxicity prediction. Among them, ToxinPred,^5^ ATSE,^7^ and ToxIBTL^8^ have been developed for peptide toxicity prediction. ToxinPred uses an ML method based on support vector machines (SVMs)^9^ and hybrid methods in combination with motif information to predict the toxicity of peptides of length at most 35. The input features for SVM training include amino acid composition (AAC), dipeptide composition (DPC), and binary profiles of patterns using one-hot encoding. Then, various SVM models are developed using one respective feature. In contrast, both ATSE and ToxIBTL use deep learning (DL) methods to predict the toxicity of peptides with 10–50 amino acids; they use the same data set. Specifically, ATSE uses a convolutional neural network (CNN)^10^ joined with bidirectional long short-term memory (BiLSTM),^11^ i.e., a CNN-BiLSTM hybrid network, to capture evolutionary information on peptides represented by a position-specific scoring matrix. It also uses a graph neural network^12^ to capture structural information represented by the molecular graph of peptides. ToxIBTL uses a CNN combined with bidirectional gated recurrent units (BiGRUs),^13^ a CNN-BiGRU hybrid network, to process the AAC, DPC, and physicochemical property features and evolutionary information represented by BLOSUM62^14^ of the input peptides. The above three predictors use SwissProt^15^ and other toxin databases to construct a positive data set. Specifically, for SwissProt, they use the keyword KW-0800 to search for toxic peptides. They all use nontoxins in SwissProt as negative samples. ToxinPred uses 1805 positive and 3593 negative samples without sequence similarity filtering as the main data set for training and other 303 positive and 300 negative samples as an independent test data set. ATSE and ToxIBTL use the same balanced peptide data set of 1932 positive and 1932 negative samples, a total of 3864 samples, with a sequence similarity of at most 90% filtered by CD-HIT^16^ for training and testing.

In this study, we present ToxTeller which provides ML-based predictors using logistic regression (LR),^17^ SVMs, random forests (RFs),^18^ and XGBoost^19^ for the toxicity prediction of peptides with 10–50 amino acids. We compile a larger data set of toxic and nontoxic peptides even with an existence evidence check for filtering out peptides with low existence evidence levels. Notably, from the compiled data set, we construct an independent test data set and a training data set, where the former shares a sequence similarity of at most 40% within itself and with the latter. To encode peptides for developing ML models, we consider composition-based and physicochemical property-based features and optimize the combination of these features. Then, we use the training data set to develop prediction models using the above four ML approaches. As suggested by many guidelines regarding the modeling for risk assessment of chemical compounds,^20−22^ it is crucial to estimate the applicability domains of derived prediction models based on the used features, where the predictions for samples outside the domain may not be reliable. Thus, we conduct analyses for applicability domain estimation using peptides in the training data set and find that 198 (99%) peptides in the independent test data set fall within the domain. Then, we evaluate the performance of ToxTeller and compare it with two existing predictors on the independent test data set. Moreover, since toxic peptides need to be avoided for vaccine and drug design because of the essential safety requirement, we analyze the false-negative predictions of toxic peptides by ToxTeller—toxic peptides falsely predicted as nontoxic—and propose strategies to reduce the number of false-negative predictions. The data sets used in the study and the codes for the trained predictors are freely available at https://github.com/comics-asiis/ToxicPeptidePrediction.

## Materials and Methods

### Data Sets

**Figure 1:**
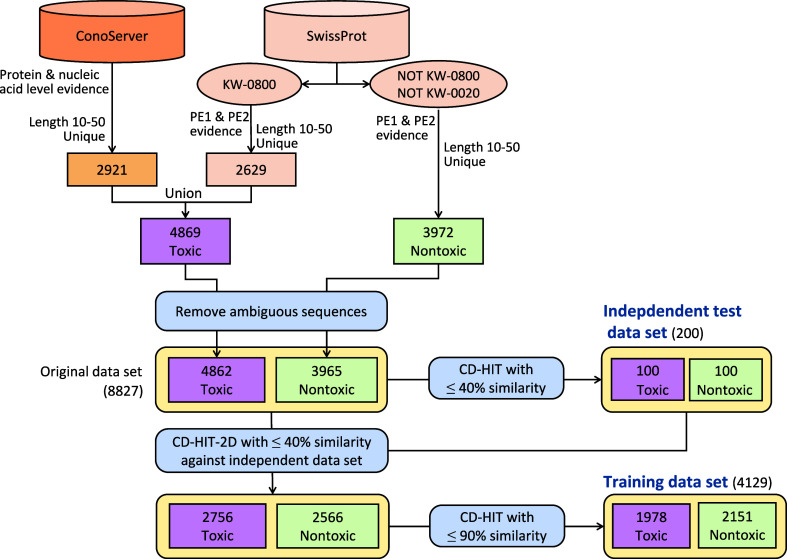
Flowchart of data set construction.

We used SwissProt (downloaded on 2023–7–25) and ConoServer^23^ (downloaded on 2023–04–12) to construct a positive data set and used only SwissProt to construct a negative data set, composed of peptides with 10–50 amino acids. From SwissProt, we compiled protein or peptide sequences having protein existence evidence at the protein (PE1) and transcript (PE2) levels for both positive and negative data. From ConoServer, we collected sequences with evidence at the protein and nucleic acid levels for positive data. Furthermore, sequences containing any unusual amino acid, e.g., X, B, were removed. The construction of our training and independent test data sets is shown in Figure 1 and described below.

To construct a positive data set, we first performed searching in SwissProt using the keyword “KW-0800” (toxin) and downloaded toxins in XML format. To obtain more positive samples, we considered not only peptides and short proteins of 10–50 amino acids but also long proteins with only one toxic region or peptide in their sequence annotations. To be specific, for a long protein containing exactly one chain (CHAIN) or one active peptide (PEPTIDE) in its annotation, if the chain or active peptide was 10–50 amino acids long and had a name annotation that was the same as the recommended name of the protein entry (RecName), it was extracted as a positive sample. We thus compiled 2629 toxic peptides with PE1 or PE2 evidence from SwissProt. Next, we collected peptides from ConoServer and filtered out redundant identical sequences. As a result, we collected 2921 toxic peptides with evidence at the protein and nucleic acid levels. Venn diagram of toxic peptides obtained from SwissProt and ConoServer is shown in Figure S1 in the Supporting Information. Combining the positive data from SwissProt and ConoServer, we obtained 4869 distinct toxic peptides.

To construct a negative data set, we used the keywords “NOT KW-0800” and “NOT KW-0020” to retrieve peptides that were neither annotated as toxins nor annotated as allergens in SwissProt and obtained a total of 3972 distinct sequences of 10–50 amino acids with PE1 or PE2 evidence. Further double-checking the positive and negative data sets, we found seven sequences that appeared in both data sets: three sequences from ConoServer and four toxic sequences in SwissProt that were identical to or overlapped with negative sequences in SwissProt under different entry names. They were then removed from both the positive and negative data sets to ensure the data set quality. In summary, we obtained an “original” data set of 8827 distinct peptides, including 4862 toxic peptides and 3965 nontoxic peptides.

To properly construct an independent test data set, we used CD-HIT (version 4.8.1) to cluster peptides with at least 40% similarity from the original data set. From the results, we randomly selected representative peptides from 200 clusters with 100 toxic and 100 nontoxic peptides as the independent test data set. Next, to construct a proper training data set, we used CD-HIT-2D with a similarity threshold of 40% to match the independent test data set against the original data set. This yielded 5322 peptides, including 2756 toxic and 2566 nontoxic peptides, that shared at most 40% similarity with the independent test data set to avoid performance overestimation. Finally, we further used CD-HIT to filter out peptides with at least 90% similarity in the 5322 peptides and obtained a training data set of 4129 peptides, consisting of 1978 toxic and 2151 nontoxic peptides for ML model development. The training data set and independent test data set can be freely accessed at https://github.com/comics-asiis/ToxicPeptidePrediction.

### Features and Encodings

To develop ML-based predictors, we consider the following three types of features to represent each input peptide.

#### Type 1: Sequence Composition-Based Features

This type of features characterizes a peptide sequence by amino acid composition and subsequences, including (1) AAC, (2) DPC, (3) 8-mer of the N-terminal (N8mer), and (4) 8-mer of the C-terminal (C8mer). The AAC feature is a vector of dimension 20, defined as the percentage of each amino acid type in the peptide. The DPC feature is defined as the percentage of each dipeptide type in the peptide, given by , where *n* is the peptide length. Note that DPC is a sparse vector of dimension 400 with at most *n* – 1 nonzero entries. The N8mer and C8mer features are used to capture the patterns of eight amino acids at both ends of the peptide using one-hot encoding, each with dimension 160.

#### Type 2: Physicochemical Property Features

We considered 14 physicochemical properties such as hydrophobicity, free energy, polarity, and volume to distinguish toxic and nontoxic peptides (Table 1); the property values of 20 amino acid types are listed in Table S1 in the Supporting Information. To check the validity of each property, we performed a two-sample *t* test (two-tailed) on peptides of the training data set. Except the polarity (Pol1) property, the other 13 properties show significant differences between toxic and nontoxic peptides and are used as features. Three different encodings are used for the 13 physicochemical properties, as illustrated in Figure S2 in the Supporting Information.

**Table 1:** Physicochemical Properties Surveyed in This Study and Their Statistical Test Results

property ID	full name	*p*-value
Hyd1	hydrophobicity^24^	8.85696 × 10^–18^
Hyd2	hydrophilicity^25^	2.93519 × 10^–5^
Hydro	hydropathy index^26^	1.93013 × 10^–17^
FEtmh	free energy for transmembrane helix^27^	4.35383 × 10^–9^
Pol1	polarity^24^	0.402536138
Pol2	polarizability^24^	2.42457 × 10^–7^
Vol	volume^28^	5.77797 × 10^–53^
VSC	volume of side chain^24^	8.78912 × 10^–57^
SA	solvent accessibility of surface area^24^	5.34114 × 10^–22^
pI	pH at isoelectric point^29^	6.65006 × 10^–39^
pKa1	p*K*_a_ of α-carboxyl group^29^	1.4229 × 10^–139^
pKa2	p*K*_a_ of α-ammonium ion^29^	1.5289 × 10^–234^
Chg	charge^30^	2.10539 × 10^–15^
NCIS	net charge index of side chain^24^	8.14791 × 10^–46^

First, the 13 physicochemical properties are encoded by a vector of dimension 13, denoted as *singPCP13*, where each property of the feature is represented by the average property value of the peptide, i.e., summing the values of the property for all of the residues in the peptide and then dividing the sum by the peptide length. Since different properties have varying scales across 20 amino acid types, e.g., [−3.4, 3] for hydrophilicity and [67.5, 237.2] for volume, the average property values of a peptide also vary greatly among the 13 properties. Then for each property, we use StandardScaler^31^ in scikit-learn^32^ (version 0.23.2) to perform *z*-score normalization on the average property values of all peptides. The *z*-score normalization is performed on the 13 properties to prevent overweighting of the properties with large values.

Second, each physicochemical property of a peptide is encoded by a vector of property values of the peptide’s residues, rather than a single summarized value as in singPCP13. To adapt to the varying value ranges of different properties, we performed *z*-score normalization on the values of 20 amino acids for each property before encoding (Table S1 in the Supporting Information). To tackle peptides of different lengths, we padded all peptides with zeros to a length of 50, the maximum peptide length, yielding a feature vector of dimension 650, denoted as *PCP13*.

Third, we selected representative physicochemical properties from the 13 properties as features, i.e., selecting a number of vectors corresponding to the selected properties from PCP13. For the selection, we calculated pairwise correlations of the 13 properties represented by singPCP13 of the peptides in the training data set. Based on the computed correlation matrix, we clustered these 13 properties into five clusters, as shown in Figure S3 in the Supporting Information. The five representative properties—free energy for transmembrane helix (FEtmh), volume (Vol), charge (Chg), p*K*_a_ of α-ammonium ion (pKa2), and p*K*_a_ of α-carboxyl group (pKa1)—of the five clusters, respectively, were selected as the feature of dimension 250, denoted by *rPCP5*, to represent input peptides.

#### Type 3: Principal Properties

As peptides can be well characterized by a number of physicochemical properties or molecular descriptors of their amino acids, principal properties are proposed to replace these individual properties and are obtained by projecting the high-dimensional property space to lower-dimensional latent spaces based on principal component analysis.^33,34^ Specifically, in these two publications, three principal properties (PPs) are reported to characterize peptides with different PP values, where each PP can be regarded as an integrative property for the 20 amino acids. Then, each PP of a peptide is encoded by a vector of property values of the peptide’s residues. In other words, each peptide is encoded by three PP vectors. Similar to PCP13 and rPCP5, all peptides encoded using the three PPs properties are padded to a length of 50 with zeros, thus yielding a feature vector of dimension 150 for each peptide. In this study, the type-3 feature PPs can be viewed as a type of feature alternative to the type-2 features.

### Hyper-Parameter Optimization of Machine Learning Approaches

We used the LR, SVM, RF, and XGBoost methods to develop prediction models. For hyper-parameter optimization, we applied a *k*-fold cross-validation technique^35^ on the training data set. However, we did not perform hyper-parameter optimization for the LR-based model, which served as a baseline for the performance of ML-based predictors. To optimize hyper-parameters for the SVM, RF, and XGBoost methods, we used PyCaret^36^ (version 2.3.10) to conduct 10-fold cross-validations using AAC + DPC + N8mer + C8mer + singPCP13 as the input features. The hyper-parameters were selected by grid search based on the highest average Matthews correlation coefficient (MCC) achieved in the 10-fold cross-validation. Details of the selection and optimized hyper-parameters are provided in Section S1 in the Supporting Information.

### Various Feature Combinations for Machine Learning

We considered various feature combinations for the performance evaluation to determine the optimized feature combination for model development. The following three feature sets were considered, i.e., using (1) composition-based type-1 features only, (2) property-based type-2 and type-3 features, (3) combined features of three type-1 features coupled with all of the type-2 and type-3 features, a total of 28 feature combinations as listed in Table 2. Specifically, Feature set 1 consists of 12 feature combinations that are comprehensive combinations of four type-1 features excluding three combinations involving N8mer + C8mer, where the feature combinations of AAC + DPC + N8mer, AAC + DPC + C8mer, and AAC + DPC + N8mer + C8mer are denoted as ADN, ADC, and ADNC, respectively.

**Table 2:** List of 28 Feature Combinations Considered for Machine Learning

feature set		feature type (s)	feature combinations
1	composition-based (12)	type-1	AAC, DPC, N8mer, C8mer; AAC + DPC, AAC + N8mer, AAC + C8mer, DPC + N8mer, DPC + C8mer; ADN^a^, ADC^b^; ADNC^c^
2	property-based (4)	type-2	singPCP13, rPCP5, PCP13
		type-3	PPs
3	combined composition- and property-based (12)	type-1 + type-2	ADN + singPCP13, ADN + rPCP5, ADN + PCP13; ADC + singPCP13, ADC + rPCP5, ADC + PCP13; ADNC + singPCP13, ADNC + rPCP5, ADNC + PCP13
		type-1 + type-3	ADN + PPs, ADC + PPs, ADNC + PPs

a ADN denotes AAC + DPC + N8mer.

b ADC denotes AAC + DPC + C8mer.

c ADNC denotes AAC + DPC + N8mer + C8mer.

### Performance Evaluation Measures

The performance of each predictor was evaluated on the independent test data set. Seven performance measures, including accuracy, precision, specificity, sensitivity, F1-score, MCC, and area under the receiver operating characteristic curve (AUC), were used to evaluate the prediction performance. Their formulas are shown in Section S2 in the Supporting Information.

## Results and Discussion

### Results of 10-Fold Cross-Validation Experiments to Determine the Optimized Feature Combination

**Figure 2:**
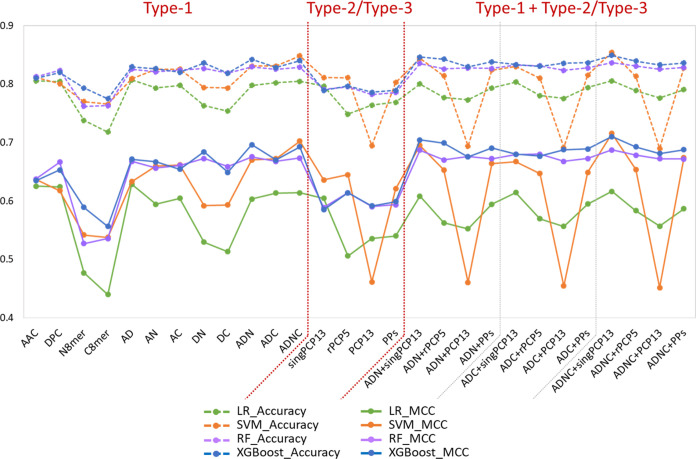
Accuracy and MCC of LR, SVM, RF, and XGBoost models based on 28 respective feature combinations in 10-fold cross-validations on training data set.

For each ML method, we conducted 28 10-fold cross-validation experiments on the training data set using the above 28 feature combinations, respectively, as the input. The detailed performance in terms of the various measures for the LR, SVM, RF, and XGBoost models is reported in Tables S2–S5 in the Supporting Information, respectively. The accuracy and MCC of these results are illustrated in Figure 2. Notably, the MCCs of prediction models based on different features vary widely. For LR, MCCs of the 28 models are in the range of 0.440–0.629. For SVM, RF, and XGBoost, MCCs are in the ranges of 0.452–0.716, 0.527–0.688, and 0.556–0.710, respectively. Denoting the difference between the highest and lowest MCC of an ML method as ΔMCC, we observe that ΔMCC = 0.189, 0.264, 0.161, and 0.154 for LR, SVM, RF, and XGBoost, respectively. It reveals that the choice of features used for developing ML models is crucial. We also note that the top four MCCs of LR models are achieved by AAC + DPC, AAC, DPC, and ADNC + singPCP13, with a slight difference of 0.013. In addition, the top three MCCs were achieved by ADNC + singPCP13, ADNC, and ADN + singPCP13 for SVM, ADNC + singPCP13, ADN + singPCP13, and ADC + rPCP5 for RF, and ADNC + singPCP13, ADN + singPCP13, and ADN + rPCP5 for XGBoost, with differences of 0.020, 0.007, and 0.011, respectively.

### Model Development for ToxTeller

To implement ToxTeller, we considered using the same features for model development of the four ML methods. We developed the ML models trained on the training data set using ADNC + singPCP13 as the input features since this feature combination achieves the highest MCC for SVM, RF, and XGBoost and the fourth highest MCC for LR with a slight difference from the top MCC in 10-fold cross-validation experiments.

Furthermore, we conducted analyses to estimate applicability domains based on the five features of ADNC + singPCP13 using the training data set and checked whether most of the peptides in the independent test data set are within the applicability domain boundaries. The details are provided in Section S3 in the Supporting Information. The results show that all of the peptides except two, i.e., 99% peptides, in the independent test data set fall within the respective domain boundaries and distribute widely in the entire domain range. This confirms the diversity of the independent test data set and supports its representativeness for performance evaluation.

### Performance Evaluation of ToxTeller

#### Performance Evaluation of LR, SVM, RF, and XGBoost Models

We first evaluated the prediction performance of ToxTeller, i.e., the LR, SVM, RF, and XGBoost models using ADNC + singPCP13 as the input features, on the independent test data set. The results are reported in Table 3. The lowest performance in all seven measures is yielded by the baseline LR model. Though the peptides of the independent test data set share at most 40% sequence similarity with the training data set, excluding the LR model, the remaining three models of ToxTeller achieve good accuracies of 0.835–0.855, sensitivities of 0.710–0.770, F1-scores of 0.811–0.842, AUCs of 0.922–0.930, and MCCs of 0.691–0.721. Among the three models, XGBoost achieves the best accuracy, sensitivity, F1-score, AUC, and MCC, and RF achieves the best precision and specificity. SVM obtains the second-best accuracy, sensitivity, F1-score, and AUC.

**Table 3:** Prediction Performance of ToxTeller on the Independent Test Data Set

predictor	accuracy	precision	sensitivity	specificity	F1-score	AUC	MCC
ToxTeller_LR	0.805	0.886	0.700	0.910	0.782	0.870	0.624
ToxTeller_SVM	0.840	0.915	0.750	0.930	0.824	0.925	0.691
ToxTeller_RF	0.835	0.947	0.710	0.960	0.811	0.922	0.692
ToxTeller_XGBoost	0.855	0.928	0.770	0.940	0.842	0.930	0.721

#### Comparison of ToxTeller with Existing Predictors

We compared the performances of ToxTeller with ML-based ToxinPred and DL-based ToxIBTL using their respective web servers with default settings. It was noted that in our independent test data set, consisting of 100 toxic and 100 nontoxic peptides, some peptides were already present in the training data sets of ToxinPred and ToxIBTL, respectively. For a fair comparison between ToxTeller and an existing predictor, we compared the performance on the independent test data set with the exclusion of peptides appearing in the respective training data set of the existing predictor. Specifically, the independent test data set contained 52 toxic and 29 nontoxic peptides of ToxIBTL’s training data set, and 32 toxic and 59 nontoxic peptides of ToxinPred’s training data set, which were excluded from comparing the performance of ToxTeller with ToxIBTL and ToxinPred, respectively. In other words, 48 toxic and 71 nontoxic peptides remained for performance comparison with ToxIBTL, and 68 toxic and 41 nontoxic peptides remained for comparison with ToxinPred. The results of comparing the performance of ToxTeller with ToxIBTL and ToxinPred are shown in Tables 4 and 5, respectively.

**Table 4:** Prediction Performance on the Subset of Independent Test Data Set Excluding Peptides in ToxIBTL’s Training Data Set^a^

predictor	accuracy	precision	sensitivity	specificity	F1-score	MCC
ToxTeller_LR	0.840	0.822	0.771	0.887	0.796	0.666
ToxTeller_SVM	0.882	0.886	0.813	0.930	0.848	0.754
ToxTeller_RF	0.891	0.949	0.771	0.972	0.851	0.776
ToxTeller_XGBoost	0.891	0.907	0.813	0.944	0.857	0.772
ToxIBTL web server	0.866	0.900	0.750	0.944	0.818	0.720

a 48 toxic peptides and 71 nontoxic peptides in the independent test data set.

**Table 5:** Prediction Performance on the Subset of Independent Test Data Set Excluding Peptides in ToxinPred’s Training Data Set^a^

predictor	accuracy	precision	sensitivity	specificity	F1-score	MCC
ToxTeller_LR	0.771	0.922	0.691	0.902	0.790	0.576
ToxTeller_SVM	0.798	0.942	0.721	0.927	0.817	0.628
ToxTeller_RF	0.761	0.938	0.662	0.927	0.776	0.574
ToxTeller_XGBoost	0.807	0.927	0.750	0.902	0.829	0.632
ToxinPred web server	0.761	0.977	0.632	0.976	0.768	0.600

a 68 toxic peptides and 41 nontoxic peptides in the independent test data set.

In comparison with ToxIBTL, ToxTeller’s RF and XGBoost models yield the same specificity and slightly higher performance by 0.007–0.063 in the remaining five measures. ToxTeller’s SVM model yields slightly better accuracy, sensitivity, F1-score, and MCC than ToxIBTL but slightly worse precision and specificity. In comparison with ToxinPred, ToxTeller’s SVM, RF, and XGBoost models achieve slightly better accuracies, sensitivities, and F1-scores by 0–0.046, 0.030–0.118, and 0.008–0.061, respectively, but worse precisions and specificities by 0.035–0.050 and 0.049–0.074, respectively. In terms of MCC, ToxTeller’s SVM and XGBoost models are slightly better by 0.028 and 0.032, respectively, but RF model slightly worse by 0.026. In summary, ToxTeller’s prediction models generally achieve slightly better performance in terms of accuracy, sensitivity, F1-score, and MCC. In summary, ToxTeller’s prediction models generally achieve slightly better performance in terms of accuracy, sensitivity, F1-score, and MCC.

### Strategies for Reducing False-Negative Predictions

#### Finding Consistent FNs of ToxTeller Predictions

For drug and vaccine design, toxic peptides should be excluded because of the essential safety concern. When a toxic peptide is predicted as toxic, i.e., a true-positive prediction, by any predictor, the peptide is very likely excluded as a therapeutic peptide candidate. However, false-negative (FN) prediction results, i.e., toxic peptides incorrectly predicted as nontoxic, need to be minimized. To conduct a rigorous examination of FN predictions, we particularly investigated toxic peptides in the independent test data set which were predicted as nontoxic by all of the four predictors of ToxTeller’s LR, SVM, RF, and XGBoost, termed as common FN predictions, i.e., not predicted as toxic by any of ToxTeller’s predictors.

Among the 100 toxic peptides in the independent test data set, the LR, SVM, RF, and XGBoost predictors using ADNC + singPCP13 features incorrectly predicted 30, 25, 29, and 23 toxic peptides as nontoxic peptides, respectively, as shown in Table S6 in the Supporting Information. Notably, 15 toxic peptides were commonly predicted as nontoxic peptides by all four models, i.e., none of the four models correctly predicted them. The remaining 85 toxic peptides in the independent test data set were correctly predicted as toxic by at least one predictor. The 15 common FNs were difficult to correctly predict; thus 15 was regarded as the baseline number of common FNs in the following analyses. We consider strategies to reduce the number of such FNs. It is worth mentioning that reducing FNs may result in an increase of false-positives (FPs), i.e., nontoxic peptides incorrectly predicted as toxic. However, compared to the increase of FNs, which is intolerable for the rigid safety concern, the increase of FPs is relatively tolerable. Thus, we still focused on investigating strategies for reducing common FNs.

#### FN Reduction by Selecting Prediction Models Based on Top Sensitivity

The prediction models used by ToxTeller were selected based on high MCC in the 10-fold cross-validation experiments using 28 respective feature combinations. To reduce common FNs, we propose using the top sensitivity in the 10-fold cross-validation experiments for model selection. As a result, the LR, SVM, and RF models achieved the top sensitivity by using the ADNC, PCP13, and ADN feature combinations, respectively. XGBoost using ADNC + singPCP13 achieved the top sensitivity, which is the same as the top-MCC model. When applying the LR, SVM, RF, and XGBoost models selected by top sensitivity for prediction on the independent test data set, 31, 19, 29, and 23 toxic peptides were incorrectly predicted as nontoxic peptides. The SVM model yielded a reduction of FNs from 25 to 19, but the LR and RF models showed no improvement in the number of FNs.

Nevertheless, note that 12 peptides were commonly predicted as nontoxic, i.e., 12 common FNs (Table S7 in the Supporting Information), by the four top-sensitivity prediction models. Among them, 10 FNs were also in the baseline 15 FNs based on high-MCC models, whereas the other two FNs were new (Figure S4 and Table S7 in the Supporting Information). The results show that using top-sensitivity prediction models yields a 20% reduction in common FNs compared with using the four high-MCC models. When avoiding FNs is a major concern, it is suggested to select the prediction model based on top sensitivity.

#### FN Reduction by Meta-Predictor Approach

The above analyses consider FNs that were incorrectly predicted by all of the ToxTeller prediction models. When such an FN is correctly predicted by any other predictor, this FN is no longer regarded as an FN. We thus propose a meta-predictor approach which combines existing predictors, such as ToxinPred and ToxIBTL, with our methods to reduce FNs.

First, combining the prediction results of ToxIBTL with ToxTeller’s results, ToxIBTL exclusively identifies eight of the baseline 15 FNs determined by ToxTeller’s predictors (Table S6 in the Supporting Information). Combining ToxIBTL thus yields a 53.33% decrease in FNs (from 15 to 7). Among the 15 baseline FNs, ToxinPred also correctly predicts two FNs, which are recovered by ToxIBTL as also shown in the table.

Second, we propose combining the prediction results of ToxIBTL with our four models selected by top sensitivity described in the previous section. From the 12 common FNs by these four models, ToxIBTL exclusively identifies four FNs and reduces the number of FNs from 12 to 8 (Table S7 in the Supporting Information), a 46.67% decrease in comparison with the baseline 15 FNs. However, this improvement yielding eight FNs is comparable to but not better than the improvement using the meta-predictor with ToxIBTL and ToxTeller’s four predictors, i.e., selected by high MCC. When combining ToxinPred with the four top-sensitivity models, two FNs are identified by ToxinPred, which are also identified by ToxIBTL.

Note that even when using a meta-server, some common FNs can still remain. For example, as shown in Figure S4 in the Supporting Information, 10 FNs consistently exist in ToxTeller’s four high-MCC-based predictors and also in the four top-sensitivity models. ToxIBTL identifies only three of the 10 FNs, still rendering seven FNs, as shown in Table S7 in the Supporting Information. The prediction results of a meta-predictor including ToxIBTL still contain at least seven FNs.

In summary, these results indicate that selecting models by top sensitivity helps to reduce FNs. Moreover, a meta-predictor using multiple classifiers including existing predictors such as ToxIBTL effectively reduces the number of incorrect predictions of toxic peptides. These two strategies can help to reduce the risk of FN prediction.

## Conclusions

In this study, we compile a relatively large data set of toxic and nontoxic peptides of length 10–50 even with checking evidence of existence by utilizing the protein annotation provided by SwissProt and ConoServer to obtain more toxic peptides for peptide toxicity prediction. Notably, from the compiled data set, we use CD-HIT and CD-HIT-2D to construct an independent test data set and a training data set, where the independent test data set shares at most 40% sequence similarity with the training data set to avoid performance overestimation. We develop ToxTeller for peptide toxicity prediction and provide four prediction models based on LR, SVM, RF, and XGBoost. To avoid using toxic peptides for vaccine and drug design, we rigorously study the FNs predicted by all of ToxTeller’s four models selected based on high MCC. To reduce the number of FNs, we suggest that selecting prediction models based on top sensitivity is a feasible strategy, which can yield fewer FNs than the high-MCC-based models. In addition, we suggest a meta-predictor approach using multiple predictors. Combining ToxTeller with the existing ToxIBTL and ToxinPred greatly reduces the number of FNs.

## Data Availability

The training and independent test data sets are freely available in the GitHub repository https://github.com/comics-asiis/ToxicPeptidePrediction. We also provide the LR, SVM, RF, and XGBoost prediction models in Pickle format and the source code for running them in the same repository.

## Supporting Information Available

Comparison of toxic peptide sequences collected from SwissProt and ConoServer (Figure S1); three different encodings of the physicochemical property features—singPCP13, PCP13, and rPCP5, using DTRPPGFTPFR as an example (Figure S2); pairwise correlations of the 13 physicochemical properties on the 4129 peptides of the training data set (Figure S3); comparison of common FNs between ToxTeller’s four predictors (trained using ADNC + singPCP13) and the four models selected by top sensitivity (Figure S4); hyper-parameter optimization for SVM, RF, and XGBoost predictors (Section S1); seven performance evaluation measures used for peptide toxicity prediction results (Section S2), and applicability domain estimation and analyses (Section S3) (PDF)
Original and normalized physicochemical property values of 20 amino acids (Table S1); performance of LR models using 28 respective feature combinations in the 10-fold cross-validations on the training data set (Table S2); performance of SVM models using 28 respective feature combinations in the 10-fold cross-validations on the training data set (Table S3); performance of RF models using 28 respective feature combinations in the 10-fold cross-validations on the training data set (Table S4); performance of XGBoost models using 28 respective feature combinations in the 10-fold cross-validations on the training data set (Table S5); false-negative predictions of toxic peptides by at least one of ToxTeller’s four models and prediction results of these peptides by ToxIBTL and ToxinPred (Table S6), and common false-negative predictions by the four top-sensitivity models and their predictions by ToxIBTL and ToxinPred (Table S7) (XLSX)
