# Supplementary material for: ToxTeller: Predicting Peptide Toxicity Using Four Different Machine Learning Approaches
Source: ACS Omega. 2024 Jul 11;9(29):32116–23. doi: 10.1021/acsomega.4c04246 (PMC11270677; doi:10.1021/acsomega.4c04246)
Supplement: Supplementary file 1 — ao4c04246_si_001.pdf [file ao4c04246_si_001.pdf]

Supporting Information

ToxTeller: Predicting Peptide Toxicity Using Four Different Machine Learning Approaches

*Jen-Hung Wang and Ting-Yi Sung**

Institute of Information Science, Academia Sinica, Taipei 11529, Taiwan

*Correspondence: tsung@iis.sinica.edu.tw

Table of Contents

Figure S1. Comparison of toxic peptide sequences collected from SwissProt and ConoServer. S3

Figure S2. Three different encodings of the physicochemical property features—singPCP13, PCP13 and rPCP5, using DTRPPGFTPFR as an example. S4

Figure S3. Pairwise correlations of the 13 physicochemical properties on the 4129 peptides of the training data set. S5

Figure S4. Comparison of common FNs between ToxTeller's four predictors (trained using ADNC+singPCP13) and the four models selected by top sensitivity. S6

Section S1. Hyper-parameter optimization for SVM, RF, and XGBoost predictors S7

Table A1. Optimized hyper-parameters for SVM, RF, and XGBoost methods S7

Section S2. Seven performance evaluation measures used for peptide toxicity prediction results ... S8

Section S3. Applicability domain estimation and analyses S9

Figure A1. Distributions of AAC of 20 amino acids in the training data set and independent test data set. S10

Figure A2. Distributions of log-distances of peptides in the training data set and independent test data set, respectively, to the center of the training data set calculated from AAC. S11

Figure A3. Distributions of log-distances of peptides in the training data set and independent test data set, respectively, to the center of the training data set calculated from DPC. S12

Figure A4. Calculation of occurrence probability of N8mer using “RIKIGLFDQLSRL” as an example. ……………………………………………………………………………...… S13

Figure A5. Distributions of log-joint frequencies of peptides in the training data set and independent test data set, respectively, calculated from (A) N8mer and (B) C8mer. …….…. S14

Figure A6. Distributions of singPCP13 values of peptides in the training data set and independent test data set, respectively, for the 13 properties. ….……………………………. S15

References ……………………………………………………………………………………… S17

Note: (1) Figures S1-S4 are supporting figures in the manuscript. Auxiliary figures shown in supporting Section S3 are labeled as Figures A1-A6, to distinguish from supporting figures in the manuscript, Figures S1-S4. For ease of reading and exposition, we keep Figures A1-A6 within the text.

(2) Auxiliary table in supporting Section S1 is labeled as Table A1, to distinguish from supporting tables in the manuscript, Tables S1-S7 shown in the supporting XLSX file.

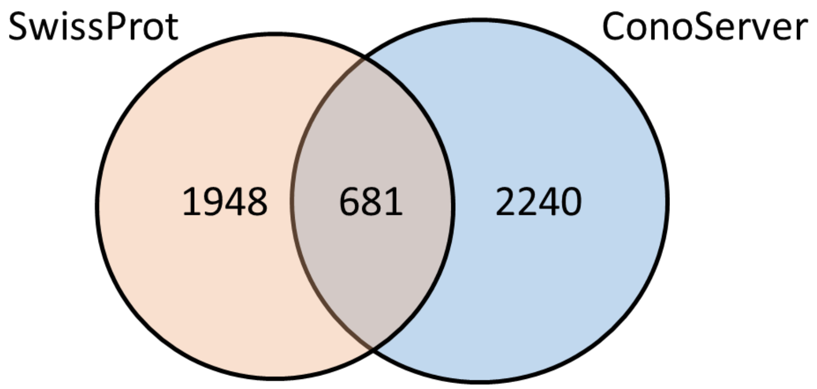

Figure S1. Comparison of toxic peptide sequences collected from SwissProt and ConoServer.

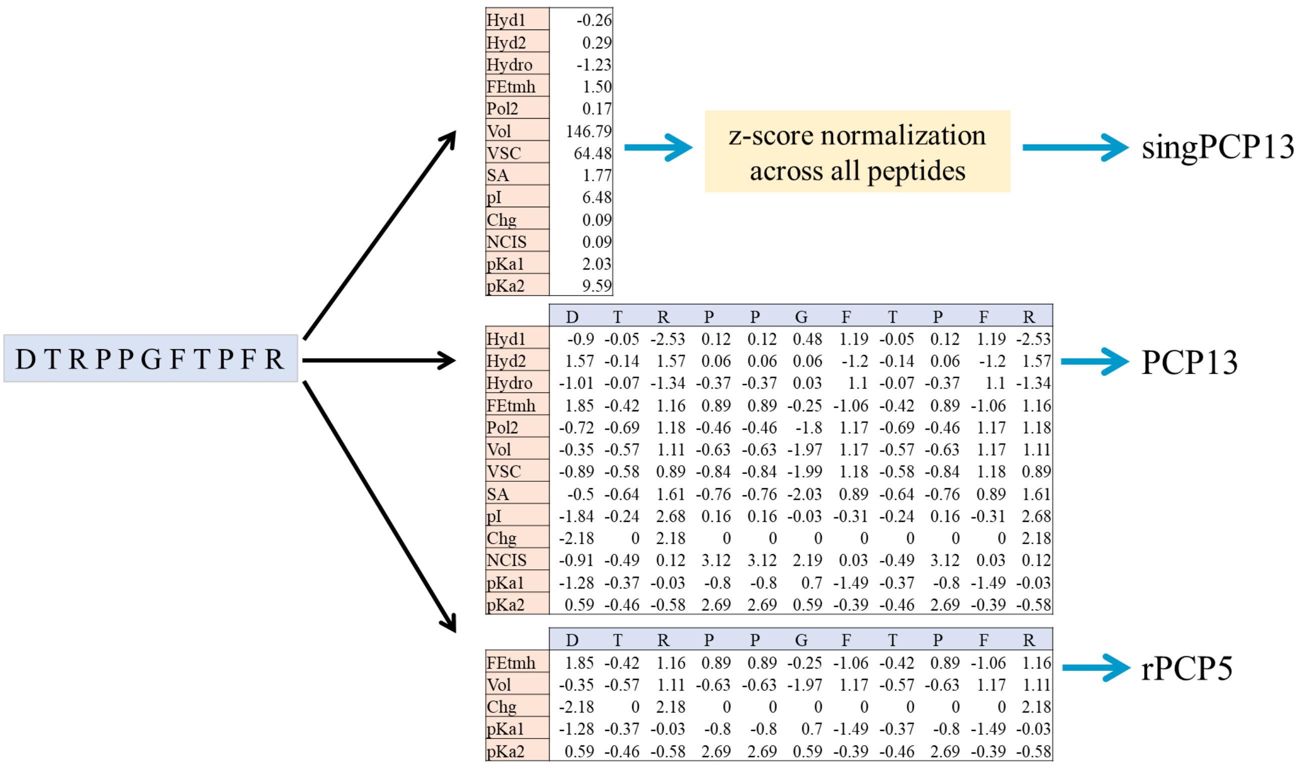

Hyd1	-0.26
Hyd2	0.29
Hydro	-1.23
FEtmh	1.50
Pol2	0.17
Vol	146.79
VSC	64.48
SA	1.77
pI	6.48
Chg	0.09
NCIS	0.09
pKa1	2.03
pKa2	9.59

	D	T	R	P	P	G	F	T	P	F	R
Hyd1	-0.9	-0.05	-2.53	0.12	0.12	0.48	1.19	-0.05	0.12	1.19	-2.53
Hyd2	1.57	-0.14	1.57	0.06	0.06	0.06	-1.2	-0.14	0.06	-1.2	1.57
Hydro	-1.01	-0.07	-1.34	-0.37	-0.37	0.03	1.1	-0.07	-0.37	1.1	-1.34
FEtmh	1.85	-0.42	1.16	0.89	0.89	-0.25	-1.06	-0.42	0.89	-1.06	1.16
Pol2	-0.72	-0.69	1.18	-0.46	-0.46	-1.8	1.17	-0.69	-0.46	1.17	1.18
Vol	-0.35	-0.57	1.11	-0.63	-0.63	-1.97	1.17	-0.57	-0.63	1.17	1.11
VSC	-0.89	-0.58	0.89	-0.84	-0.84	-1.99	1.18	-0.58	-0.84	1.18	0.89
SA	-0.5	-0.64	1.61	-0.76	-0.76	-2.03	0.89	-0.64	-0.76	0.89	1.61
pI	-1.84	-0.24	2.68	0.16	0.16	-0.03	-0.31	-0.24	0.16	-0.31	2.68
Chg	-2.18	0	2.18	0	0	0	0	0	0	0	2.18
NCIS	-0.91	-0.49	0.12	3.12	3.12	2.19	0.03	-0.49	3.12	0.03	0.12
pKa1	-1.28	-0.37	-0.03	-0.8	-0.8	0.7	-1.49	-0.37	-0.8	-1.49	-0.03
pKa2	0.59	-0.46	-0.58	2.69	2.69	0.59	-0.39	-0.46	2.69	-0.39	-0.58

	D	T	R	P	P	G	F	T	P	F	R
FEtmh	1.85	-0.42	1.16	0.89	0.89	-0.25	-1.06	-0.42	0.89	-1.06	1.16
Vol	-0.35	-0.57	1.11	-0.63	-0.63	-1.97	1.17	-0.57	-0.63	1.17	1.11
Chg	-2.18	0	2.18	0	0	0	0	0	0	0	2.18
pKa1	-1.28	-0.37	-0.03	-0.8	-0.8	0.7	-1.49	-0.37	-0.8	-1.49	-0.03
pKa2	0.59	-0.46	-0.58	2.69	2.69	0.59	-0.39	-0.46	2.69	-0.39	-0.58

Figure S2. Three different encodings of the physicochemical property features—singPCP13, PCP13 and rPCP5, using DTRPPGFTPFR as an example. The singPCP13 encoding uses the average property value of the peptide for each property and then performs z-score normalization across all peptides. For PCP13 and rPCP5, we first perform z-score normalization on the values of 20 amino acids for each property and then use the normalized property values for encoding. The PCP13 and rPCP5 encodings are padded to length of 50 with zeroes (omitted in the figure).

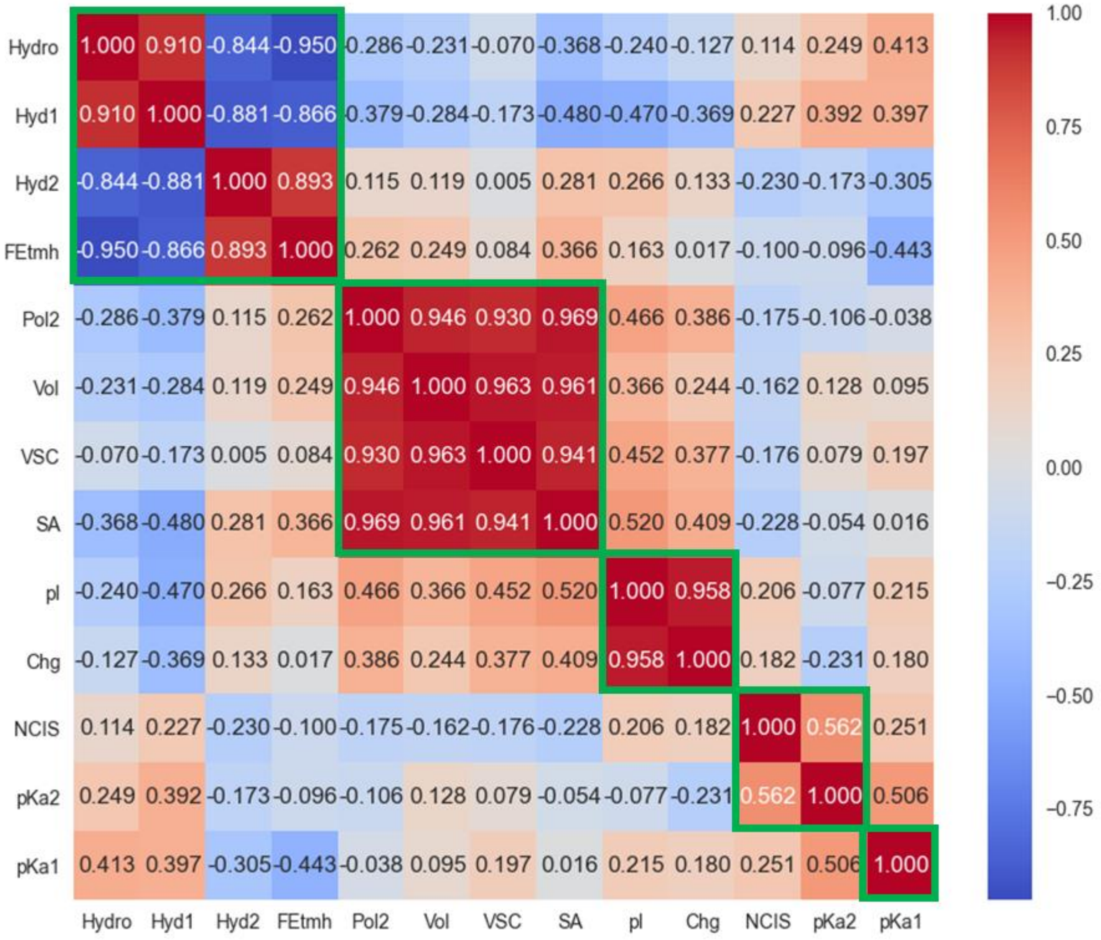

Figure S3. Pairwise correlations of the 13 physicochemical properties on the 4129 peptides of the training data set. These 13 physicochemical properties are clustered into five clusters, where FEtmh (Free energy for transmembrane helix), Vol (Volume), Chg (Charge), pKa1 (pKa of α-carboxyl group), and pKa2 (pKa of α-ammonium ion) are selected from each cluster to represent each peptide, denoted as rPCP5.

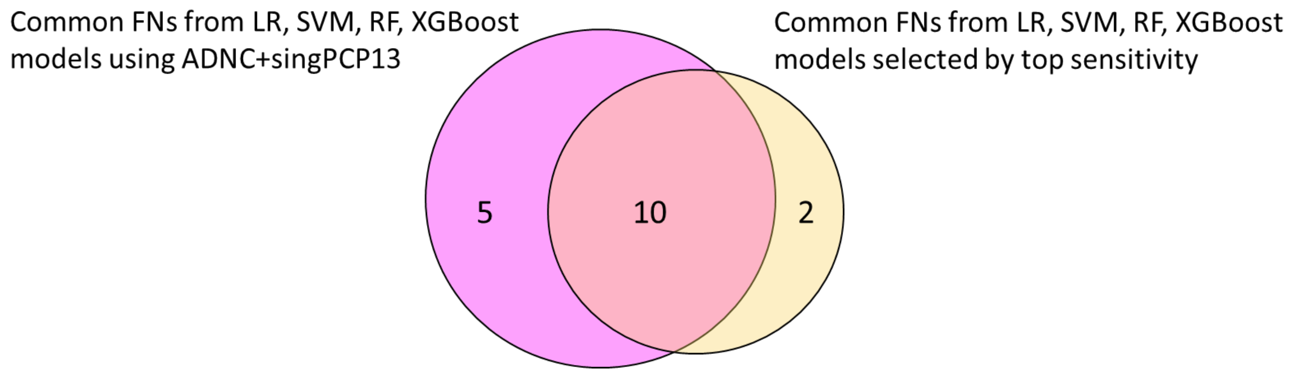

Figure S4. Comparison of common FNs between ToxTeller's four predictors (trained using ADNC+singPCP13) and the four models selected by top sensitivity.

Section S1. Hyper-parameter optimization for SVM, RF, and XGBoost predictors

To optimize the hyper-parameters of our SVM, RF, and XGBoost models, we performed a grid search for each model using 10-fold cross-validation (CV) on the training data set, with the input features AAC+DPC+N8mer+C8mer+singPCP13. The hyper-parameters were then selected based on the highest average Matthews correlation coefficient (MCC) across the 10 folds. The procedure was conducted using PyCaret[1] (version 2.3.10).

For SVM using the radial basis function kernel, with the penalty parameter (C) of 1–50 and the kernel width parameter (γ) of 0.0001–1, C= 20 and γ= 0.01 were selected (as shown in Table A1).

For RF, we considered the following parameters: either gini or entropy criterion, max_depth of 5–10, min_samples_leaf of 1–5, min_samples_split of 2–6, and n_estimators of 30–100. Gini criterion, max_depth= 9, min_samples_leaf= 1, min_samples_split= 3, and n_estimators= 30 were selected.

For XGBoost, we considered the following parameters: a learning_rate of 0.1–0.4, max_depth of 5–8, subsample of 0.7–1.0, colsample_bytree of 0.7–1.0, and n_estimators of 50–110. As a result, learning_rate= 0.1, max_depth= 8, subsample= 0.9, colsample_bytree= 0.9, and n_estimators= 50 were selected.

Table A1. Optimized hyper-parameters for SVM, RF, and XGBoost methods

Method	Parameters (default if not mentioned here)
SVM	C: 20<br>γ: 0.01
RF	criterion: gini<br>max_depth: 9<br>min_samples_leaf: 1<br>min_samples_split: 3<br>n_estimators: 30
XGBoost	learning_rate: 0.1<br>max_depth: 8<br>subsample: 0.9<br>colsample_bytree: 0.9<br>n_estimators: 50

Section S2. Seven performance evaluation measures used for peptide toxicity prediction results

Seven performance measures, including accuracy, precision, specificity, sensitivity, F1-score, MCC, and area under the receiver operating characteristic curve (AUC), were used to evaluate the prediction performance. Their formulae are shown as follows:

$$Accuracy = \frac{TP + TN}{TP + TN + FP + FN}$$

$$Precision = \frac{TP}{TP + FP}$$

$$Specificity = \frac{TN}{TN + FP}$$

$$Sensitivity = \frac{TP}{TP + FN}$$

$$F1\text{-}score = \frac{2 \times Precision \times Sensitivity}{Precision + Sensitivity}$$

$$MCC = \frac{TP \times TN - FP \times FN}{\sqrt{(TP+FP) \times (TP+FN) \times (FP+TN) \times (TN+FN)}}$$

where TP, TN, FP, and FN stand for the numbers of true positives, true negatives, false positives, and false negatives, respectively, in binary classification.

To calculate AUC, we examined all possible pairs between positive samples (S_P) and negative samples (S_N), and scored each pair by the concordance of their scores provided by the model as follows:

$$Concordance(i,j) = \begin{cases} 1 & if \ \ Score(P_i) > Score(N_j) \\ 0.5 & if \ \ Score(P_i) = Score(N_j) \\ 0 & if \ \ Score(P_i) < Score(N_j) \end{cases}$$

where Pi is the ith positive sample and Nj is the jth negative sample, and calculated AUC as follows:[2]

$$AUC = \frac{\sum_{i=1}^{S_P} \sum_{j=1}^{S_N} Concordance(i,j)}{S_P \times S_N} \ .$$

Section S3. Applicability domain estimation and analyses

To verify the applicability of our prediction models, we conducted analyses to estimate applicability domains (ADs) based on the five features of ADNC+singPCP13, i.e., AAC+DPC+N8mer+C8mer+singPCP13, using the training data set and checked how many peptides of the independent test data set are out of the AD boundaries. If a large percentage of peptides in the test data set are out of the AD boundaries, the reliability of prediction results of using the prediction models on the test data set is limited.

To estimate ADs of the five features of ADNC+singPCP13 used to develop prediction models based on the training data set, we proposed different measures for estimation as described below.

(1) AAC (amino acid composition)

We analyzed the respective distributions of AAC of 20 amino acids in the training data set and independent test data set. Figure A1 show the distributions of AACs of 20 amino acid types.

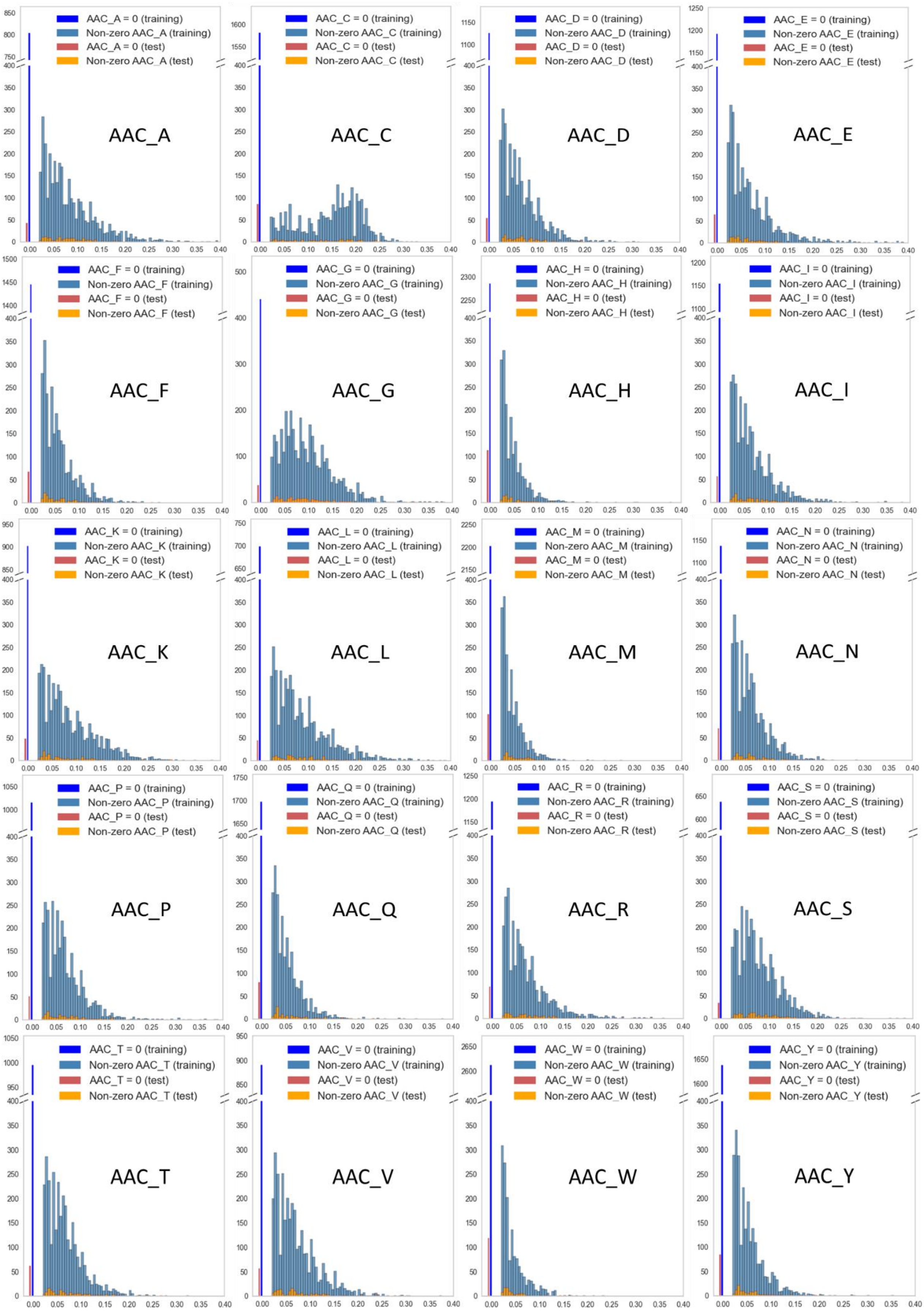

Figure A1. Distributions of AAC of 20 amino acids in the training data set and independent test data set.

Since the distribution for each amino acid has a large number of zeros and is not normal, we proposed a Euclidean distance measure for each peptide in the training data set (4129 peptides) to the remaining 4128 peptides to estimate AD. First, each peptide is represented by a peptide AAC-vector, a dim-20 vector consisting of AACs of 20 amino acids in the peptide. Given a peptide in the training data set, the remaining 4128 peptides are represented by their center, calculated by averaging their peptide AAC-vectors. Then we calculate the Euclidean distance between the given peptide and the center. Iteratively, all the peptides in the training data set yield 4129 distances. We perform a log transformation on these distances to derive 4129 log-distances, forming a reference distribution for AD estimation. As shown in Figure A2, the minimum and maximum values of the reference distribution are -2.3553 and -0.3481, respectively, defining the AD boundaries for the AAC feature.

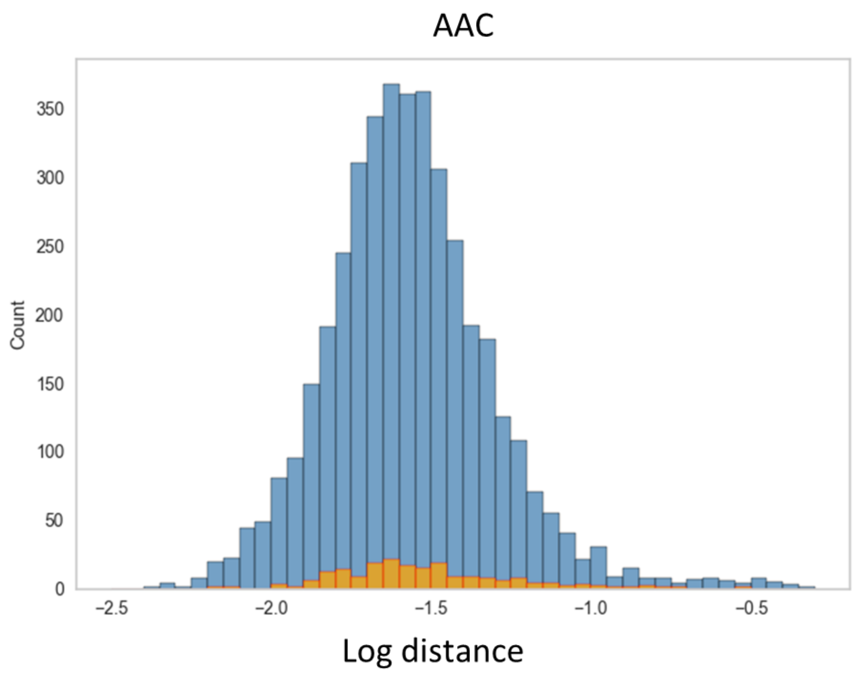

Figure A2. Distributions of log-distances of peptides in the training data set (blue bars) and independent test data set (orange bars), respectively, to the center of the training data set calculated from AAC.

To assess a peptide in the independent test data set, we calculate the Euclidean distance of the peptide to the entire training data set represented by the center of 4129 peptides. The log-distances of 200 peptides in the independent test data set are shown in Figure A2. The results reveal that all the peptides in our independent test data set are inside the AD of AAC.

(2) DPC (dipeptide composition)

For AD estimation of DPC, we apply an approach similar to that of AAC. Instead of an AAC-vector,

each peptide in the training data set is represented by a dim-400 DPC-vector consisting of its DPCs of 400 dipeptides to calculate its Euclidean distance to the remaining 4128 peptides. After the log transformation on the resulting 4129 distances, we obtained the reference distribution consisting of 4129 log-distances, as depicted in Figure A3. The reference distribution has values ranging from -2.0483 to -0.5577, defining the AD boundaries for the DPC feature. We also assess the log-distances of 200 peptides in the independent test data set, as depicted in Figure A3. The results show that all the peptides in our independent test data set are inside the AD of DPC.

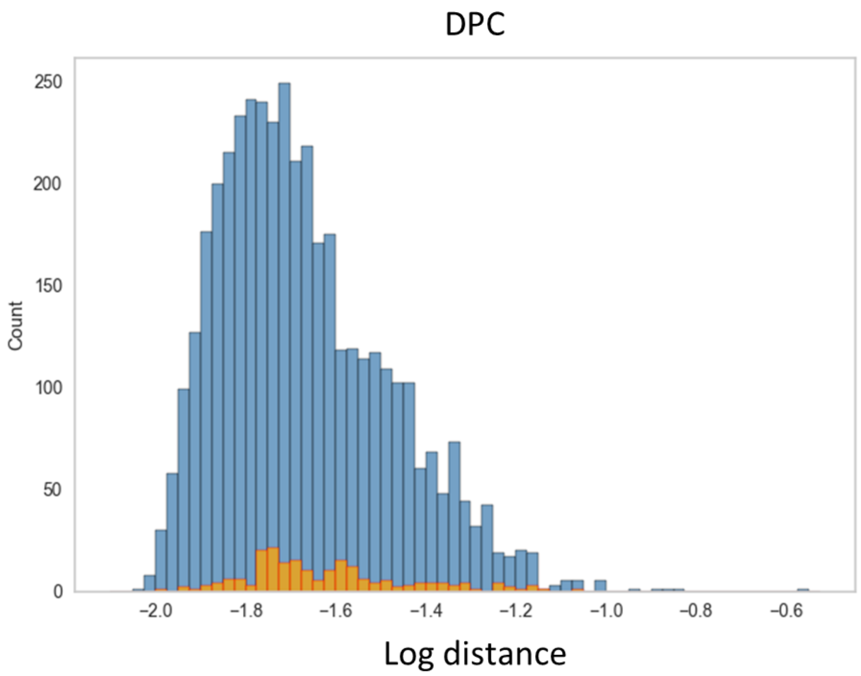

Figure A3. Distributions of log-distances of peptides in the training data set (blue bars) and independent test data set (orange bars), respectively, to the center of the training data set calculated from DPC.

(3) N8mer (the 8-mer of the N-terminal)

Since N8mer is encoded by one-hot encoding as a dim-160 binary vector, the approach using Euclidean distance to measure the spread of a peptide in the training data set with the remaining peptides is not appropriate. Instead, we define a joint frequency as the measure for each single peptide in the training data set. Given a peptide, we first generate a 20×8 count table with 20 rows representing 20 amino acid types and 8 columns representing 8 positions of the N-terminal to record the numbers of amino acid occurrences at the respective positions based on the remaining 4128 peptides. Next, we convert these 160 counts into frequencies, i.e., dividing each entry by 4128, to generate a frequency table. Then the occurrence probability of N8mer of the given peptide can be estimated by the joint frequency, i.e., multiplying the eight frequencies from the table corresponding to the amino acids of N8mer. Figure A4 illustrates the frequency table and the calculation of occurrence probability of N8mer using the

peptide “RIKIGLFDQLSRL” as an example.

Given peptide: RIKIGLFDQLSRL

Table of occurrence frequencies:

	1	2	3	4	5	6	7	8
A	0.101	0.071	0.061	0.058	0.054	0.066	0.065	0.071
C	0.045	0.092	0.084	0.071	0.051	0.068	0.063	0.085
D	0.060	0.062	0.071	0.064	0.055	0.045	0.046	0.051
E	0.044	0.047	0.063	0.079	0.061	0.062	0.065	0.063
F	0.035	0.039	0.031	0.032	0.050	0.047	0.034	0.036
G	0.156	0.047	0.079	0.087	0.079	0.096	0.087	0.087
H	0.014	0.009	0.013	0.013	0.017	0.017	0.018	0.014
I	0.029	0.065	0.054	0.044	0.053	0.066	0.037	0.039
K	0.036	0.062	0.058	0.067	0.061	0.050	0.093	0.073
L	0.032	0.107	0.065	0.069	0.084	0.086	0.077	0.056
M	0.161	0.012	0.013	0.011	0.014	0.013	0.012	0.032
N	0.033	0.033	0.036	0.039	0.053	0.039	0.037	0.042
P	0.011	0.048	0.077	0.067	0.066	0.060	0.052	0.044
Q	0.042	0.023	0.027	0.038	0.024	0.035	0.032	0.036
R	0.026	0.038	0.047	0.045	0.053	0.047	0.039	0.046
S	0.071	0.070	0.060	0.082	0.066	0.058	0.090	0.082
T	0.040	0.058	0.058	0.049	0.052	0.049	0.055	0.060
V	0.040	0.079	0.057	0.043	0.063	0.056	0.052	0.044
W	0.009	0.020	0.024	0.010	0.014	0.019	0.018	0.016
Y	0.017	0.017	0.022	0.033	0.030	0.021	0.027	0.024

Occurrence probability of N8mer of the peptide:

$0.026 \times 0.065 \times 0.058 \times 0.044 \times 0.079 \times 0.086 \times 0.034 \times 0.051$

Figure A4. Calculation of occurrence probability of N8mer using “RIKIGLFDQLSRL” as an example.

Iteratively, all the peptides in the training data set yield 4129 joint frequencies of N8mers. We apply a log transformation on these joint frequencies to yield 4129 log-joint frequencies, forming a reference distribution for AD estimation, as depicted in Figure A5(A). Then the AD boundaries of N8mer are defined by the minimum and maximum of the reference distribution, -29.4196 and -19.0341, respectively.

To assess a peptide in the independent test data set, we calculate the log-joint frequency of the peptide, where the frequency table is derived from the 4129 peptides in the training data set. The log-joint frequencies of 200 peptides in the independent test data set are shown in Figure A5(A). The results show that all the peptides in our independent test data set are inside the AD of N8mer.

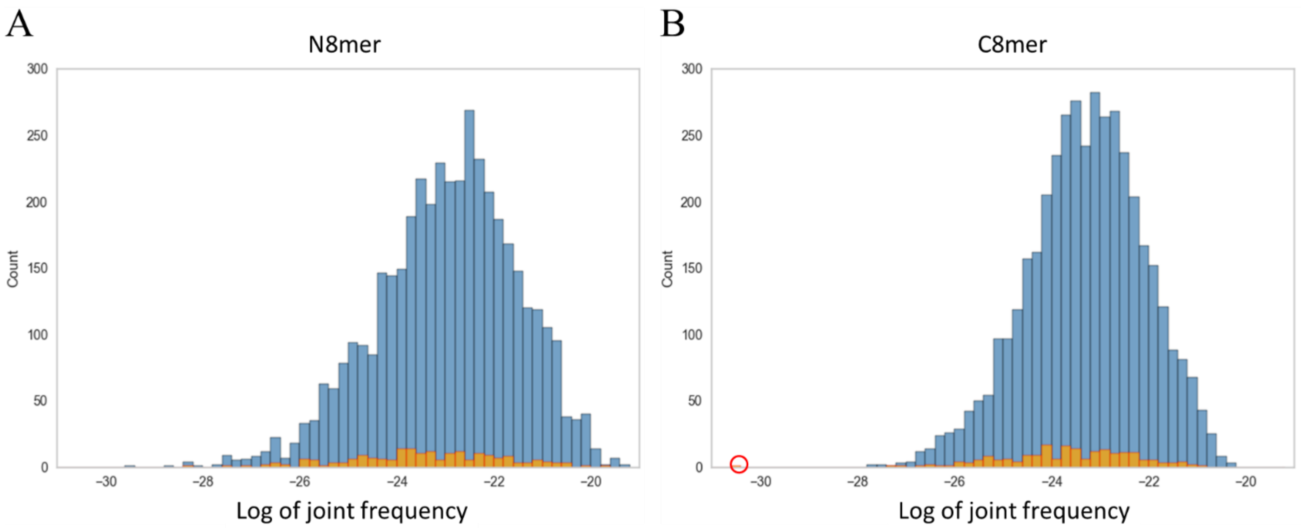

Figure A5. Distributions of log-joint frequencies of peptides in the training data set (blue bars) and independent test data set (orange bars), respectively, calculated from (A) N8mer and (B) C8mer. The red circle in (B) highlights the peptide outside the AD boundaries of C8mer.

(4) C8mer (the 8-mer of the C-terminal)

For AD estimation of C8mer, we adopt a procedure similar to that for N8mer to derive the frequency table for calculating the joint frequency of C8mer. The resulting reference distribution of C8mer is shown in Figure A5(B). The AD boundaries, defined by the minimum and maximum of the distribution, are -27.7981 and -20.2804, respectively. We assess the 200 peptides in the independent test data set and find only one peptide “DWTYTTWLWWWWWWT” falling outside the AD boundaries.

(5) singPCP13

The singPCP13 feature is a dim-13 vector, where each entry corresponds to the average of a physicochemical property of the peptide. For each property, we can obtain its distribution of the peptides in the training data set and independent test data set, respectively. Defining the AD boundaries by the minimum and maximum values of each property’s distribution obtained from the training data set, we assess the peptides in the independent test data set. All the distributions of the 13 physicochemical properties are shown in Figure A6. In total, there are only two peptides in the independent test data set falling outside the AD boundaries for some specific properties. First, the peptide “MRAKWRKKRMRRLKRKRRKMRQRSK” is outside the AD boundaries of Pol2, VSC, and SA; second, the peptide “DWTYTTWLWWWWWWT” is outside the AD boundaries of Pol2, Vol, VSC, pKa1 and Hyd2. The remaining 198 (99%) peptides in the independent test data set fall within the AD boundaries of the 13 physicochemical properties.

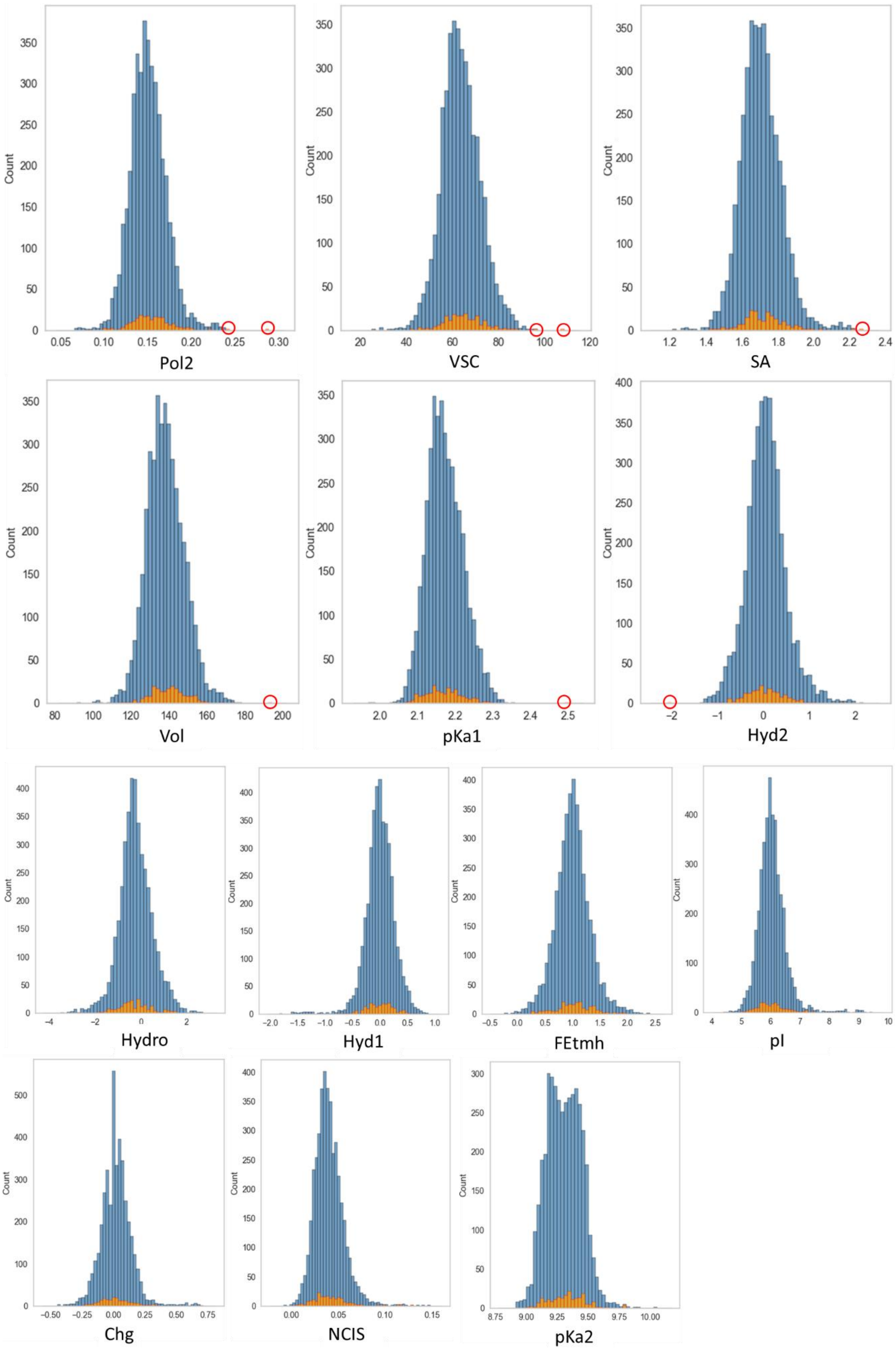

Figure A6. Distributions of singPCP13 values of peptides in the training data set (blue bars) and independent test data set (orange bars), respectively, for the 13 properties. Red circles highlight peptides outside the AD boundaries.

In summary, the above AD analyses for the five features show that the peptides in the independent test data set reveal a distribution spanning the entire respective AD range. Moreover, 99% of the peptides in the independent test data set fall within the AD boundaries.

References

(1) Ali, M. PyCaret: An Open Source, Low-Code Machine Learning Library in Python, 2023 https://www.pycaret.org (accessed December 2023).

(2) Hanley, J. A.; McNeil, B. J. The Meaning and Use of the Area Under a Receiver Operating Characteristic (ROC) Curve. *Radiology* **1982**, *143*, 29–36.
